# A prospective investigation of interleukin-8 levels in pediatric acute respiratory failure and acute respiratory distress syndrome

**DOI:** 10.1186/s13054-019-2342-8

**Published:** 2019-04-17

**Authors:** Heidi Flori, Anil Sapru, Michael W. Quasney, Ginny Gildengorin, Martha A. Q. Curley, Michael A. Matthay, Mary K. Dahmer, for the BALI and RESTORE Study Investigators, Pediatric Acute Lung Injury and Sepsis Investigators (PALISI) Network, Scot T. Bateman, M. D. Berg, Santiago Borasino, G. Kris Bysani, Allison S. Cowl, Cindy Darnell Bowens, E. Vincent S. Faustino, Lori D. Fineman, A. J. Godshall, Ellie Hirshberg, Aileen L. Kirby, Gwenn E. McLaughlin, Shivanand Medar, Phineas P. Oren, James B. Schneider, Adam J. Schwarz, Thomas P. Shanley, Lauren R. Sorce, Edward J. Truemper, Michele A. Vander Heyden, Kim Wittmayer, Athena Zuppa, David Wypij

**Affiliations:** 1 Division of Pediatric Critical Care Medicine, Department of Pediatrics and Communicable Diseases, University of Michigan, 1500 East Medical Center Dr, F6790/5243, Ann Arbor, MI 48109 USA; 2 Department of Pediatrics, University of California, Los Angeles, CA USA; 3 Children’s Hospital Oakland Research Institute, UCSF Benioff Children’s Hospitals, Oakland, CA USA; 4 Department of Family and Community Health (School of Nursing), Division of Anesthesia and Critical Care Medicine (Perelman School of Medicine), University of Pennsylvania, Philadelphia, PA USA; 5 Research Institute, Children’s Hospital of Philadelphia, Philadelphia, PA USA; 6 Departments of Medicine and Anesthesia, Cardiovascular Research Institute, University of California, San Francisco, CA USA

**Keywords:** Biomarkers, Acute respiratory distress syndrome, Genetic variants, Critical illness, ARDS, PARDS, SNP

## Abstract

**Background:**

The association of plasma interleukin-8 (IL-8), or IL-8 genetic variants, with pediatric acute respiratory distress syndrome (PARDS) in children with acute respiratory failure at risk for PARDS has not been examined. The purpose of this study was to examine the association of early and sequential measurement of plasma IL-8 and/or its genetic variants with development of PARDS and other clinical outcomes in mechanically ventilated children with acute respiratory failure.

**Methods:**

This was a prospective cohort study of children 2 weeks to 17 years of age with acute airways and/or parenchymal lung disease done in 22 pediatric intensive care units participating in the multi-center clinical trial, Randomized Evaluation of Sedation Titration for Respiratory Failure (*RESTORE*). Plasma IL-8 levels were measured within 24 h of consent and 24 and 48 h later. DNA was purified from whole blood, and IL-8 single nucleotide polymorphisms, rs4073, rs2227306, and rs2227307, were genotyped.

**Results:**

Five hundred forty-nine patients were enrolled; 480 had blood sampling. Plasma IL-8 levels ranged widely from 4 to 7373 pg/mL. Highest IL-8 levels were observed on the day of intubation with subsequent tapering. Levels of IL-8 varied significantly across primary diagnoses with the highest levels occurring in patients with sepsis and the lowest levels in those with asthma. Plasma IL-8 was strongly correlated with oxygenation defect and severity of illness. IL-8 was consistently higher in PARDS patients compared to those without PARDS; levels were 4–12 fold higher in non-survivors compared to survivors. On multivariable analysis, IL-8 was independently associated with death, duration of mechanical ventilation, and PICU length of stay on all days measured, but was not associated with PARDS development. There was no association between the IL-8 single nucleotide polymorphisms, rs4073, rs2227306, and rs2227307, and PARDS development or plasma IL-8 level.

**Conclusions:**

When measured sequentially, plasma IL-8 was robustly associated with multiple, relevant clinical outcomes including mortality, but was not associated with PARDS development. The wide range of plasma IL-8 levels exhibited in this cohort suggests that further study into the heterogeneity of this patient population and its impact on individual responses to PARDS treatment is warranted.

**Electronic supplementary material:**

The online version of this article (10.1186/s13054-019-2342-8) contains supplementary material, which is available to authorized users.

## Background

Approximately 3% of children admitted to the pediatric intensive care unit are diagnosed with pediatric acute respiratory distress syndrome (PARDS) [1], which currently carries a mortality rate of 17–51% in children [1–4] and 35–60% in adults [5, 6]. The pathogenesis of both adult and pediatric ARDS (PARDS) involves the release of pro-inflammatory cytokines and injury to both the lung vascular endothelium and alveolar epithelium. As damage progresses, proteins leak into the alveolar space, further stimulating the influx of neutrophils and macrophages into the area, propagating the inflammatory response [7, 8]. The influx of neutrophils is triggered by the increased expression and release of the chemoattractant, interleukin-8 (IL-8). The expression of IL-8 is stimulated by the classic pro-inflammatory cytokines tumor necrosis factor (TNF) and IL-1 which are released early in the inflammatory response. IL-8 is produced by multiple cell types including, but not limited to, monocytes, macrophages, fibroblasts, and endothelial cells [9]. Plasma IL-8 levels are almost immeasurable in the healthy child and are specifically elevated under conditions which trigger inflammation [8, 10–15].

In adults with ARDS, elevated levels of IL-8 in both plasma and bronchoalveolar lavage fluid have been associated with increased risk of death and multiple organ system failures [16–18]. In pediatrics, initial investigations indicate that elevations in plasma IL-8 measured at the start of PARDS correlate with worse clinical outcomes and mortality [8]. The association of IL-8 with outcomes in children with acute respiratory failure at risk for PARDS, or on multiple days after established PARDS, has not been examined, nor has the association of genetic variants of IL-8 with PARDS been examined. We hypothesized that plasma IL-8 or specific IL-8 variants would be associated with PARDS and with worse clinical outcomes both in children at risk for PARDS and in children with PARDS. The objective of this study was to determine whether plasma IL-8 levels, measured on sequential days in pediatric patients with acute respiratory failure, can identify children at increased risk of developing PARDS or correlate with clinically relevant outcomes in children with acute respiratory failure.

## Methods

This study, Genetic Variation and Biomarkers in Children with Acute Lung Injury (BALI), was an ancillary study to the multi-site clinical trial, Randomized Evaluation of Sedation Titration for Respiratory Failure (RESTORE; U01 HL086622) [19]. BALI was designed to examine the association of specific plasma protein and genetic biomarkers with PARDS in prospectively enrolled children with acute respiratory failure. Details of the study methodology have been published previously [20]. Twenty-two of the 31 pediatric intensive care units (PICUs) participating in RESTORE also participated in this study. The study was approved by the Institutional Review Boards at all participating sites. Blood samples were taken within 24 h of consent and again 24 and 48 h later. Plasma IL-8 was assayed in duplicate by ELISA (R&D Systems, #D800C). The reported limit of detection of the assay is 7.5 pg/ml; intra- and inter-assay coefficients of variations are reported to be 5.8% and 7.7%, respectively.

The primary analyses examined the association of plasma or genetic biomarkers with the presence of PARDS. For plasma IL-8, analyses examining the association with the presence of PARDS were done both across days and on each individual day. PARDS was defined using oxygenation index (OI) or oxygen saturation index (OSI) as described by the Pediatric Acute Lung Injury Consensus Conference [3] except that all patients defined as having PARDS also had bilateral infiltrates within 2 days before or 1 day after meeting OI or OSI criteria for PARDS. These chest radiograph criteria were applied as RESTORE and BALI were both conceived when the American European Consensus Conference (AECC) definition [21] was used to identify PARDS. Consequently, chest radiograph data only included the presence or absence of bilateral infiltrates. Individual chest radiographs were not collected as part of either study. Secondary analyses examined the association of biomarkers with oxygenation defect (OI, PaO_2_/FiO_2_) and other relevant clinical outcomes including duration of mechanical ventilation, PICU length of stay in survivors, and 90-day in-hospital mortality. Duration of mechanical ventilation was defined as done in RESTORE [19] with patients assigned 28 days if they remained intubated or were transferred or died prior to day 28, therefore making this outcome equivalent to ventilator-free days. Evaluation of the impact of the primary diagnosis associated with acute respiratory failure was conducted in two ways. First, diagnostic categories were evaluated based on categories reported in the parent RESTORE trial (bronchiolitis, aspiration, sepsis, pneumonia, asthma, and others). In addition, the primary diagnoses were trichotomized into infectious (bronchiolitis, laryngotracheobronchitis, pertussis, pneumonia, sepsis), non-infectious (thoracic trauma, aspiration, edema, and transfusion-associated), and indeterminant (asthma, chronic lung disease, acute respiratory failure post bone marrow transplantation, pulmonary hemorrhage, and acute chest syndrome) categories.

### Statistics

The statistical analysis was performed using SAS version 9.4. Development of PARDS, death, duration of mechanical ventilation, and length of PICU stay in survivors were the outcomes examined for association with plasma IL-8 levels across all days and if significant with IL-8 levels on each individual day. Due to the skewed distribution of plasma IL-8 levels, a natural log transformation was used for the analyses. Basic univariate (including frequency distributions or means, medians and standard deviations, analysis of variance, Wilcoxon rank-sum tests) and bivariate (including chi-square, Fisher’s exact, *t* tests, Pearson correlations, or longitudinal models, as appropriate) analyses of all demographic measures, clinical measures, and outcomes were conducted across all study days and on each individual study day (0, 1, 2, and 3). Comparisons with respect to these variables used generalized estimating equations (GEE) to account for correlations within individuals. Study hypotheses were evaluated using longitudinal models across days and if significant, with separate models for each day using logistic regression models for binary outcomes, Poisson regression models for the duration of mechanical ventilation, and linear regression for the length of PICU stay. The results of the bivariate analyses and forward stepwise linear models were used to develop the final multivariate models. The potential covariates of age, gender, race, ethnicity, prior medical co-morbidity (including the history of prematurity, asthma, seizure disorder, neurological disorder, cancer, or immune deficiency), primary diagnosis associated with acute respiratory failure, and PRISM III score were considered for the multivariate models. For the final models, each outcome was modeled as a function of age, PRISM III score, diagnosis of sepsis, and IL-8 level, with study day included when longitudinal models across all days were evaluated. The diagnosis of sepsis was correlated to a past medical history of cancer and of immune deficiency; therefore, only sepsis was included in the final model. All multivariable logistic regression models of the binary outcomes accounted for individual clustering using GEE and adjusted for the covariates to assess the association of IL-8 across all days with outcomes. Final models for each individual day included IL-8 as a fixed covariate, whereas when examining IL-8 across all days, IL-8 was included as a time-varying covariate. The GEE analysis across all days included only patients with two (*n* = 143) or three (*n* = 208) IL-8 measurements within the time frame of interest; 73 patients with only one measurement were not included in the analysis. The total number of patients included in analyses done on each day is indicated in the figures, or is shown in table legends, and includes the 73 patients that were dropped from the analyses done across all days. We reported odds ratios with 95% confidence intervals. All statistical tests were two-tailed, with a significance level of 0.05.

### Genotyping and genetic association analysis

DNA was extracted from whole blood using the Wizard Genomic DNA Purification Kit (Promega, Madison, WI). Ninety-two percent of the samples (*n* = 481) had sufficient DNA for genotyping which was done using a custom Illumina Golden gate panel which included the three IL-8 SNPs of interest, rs4073, rs2227306, and rs2227307. SNP call rates were 100%, and genotypes did not deviate from Hardy-Weinberg equilibrium. Genotyping was done blinded to the clinical status of individuals. The association of these genetic variants with PARDS was examined as described previously [20]. Briefly, the cohort was stratified by race and ethnicity into three major subgroups, non-Hispanic Caucasians, Hispanic Caucasians, and African Americans (52%, 20%, and 17% of the cohort, respectively). Association of IL-8 SNPs with PARDS within each group was examined, and then, results of the individual subgroups were combined and examined by meta-analysis using the program METAL [22].

## Results

Five hundred forty-nine patients were enrolled in the study with 480 having plasma samples. Clinical characteristics of the entire BALI cohort as well as those with and without PARDS have been described previously [20]. In brief, the patients enrolled in BALI were of mixed race and ethnicity with the major groups being non-Hispanic White (52%, *n* = 287), Hispanic White (20%, *n* = 113), and African American (17%, *n* = 92). The most frequent primary diagnoses associated with acute respiratory failure were pneumonia (37%, *n* = 203), bronchiolitis (20%, *n* = 107), and acute respiratory failure related to sepsis (19%, *n* = 104). Prior medical co-morbidities included history of asthma (16%, *n* = 89), prematurity (13%, *n* = 72), seizure disorder (10%, *n* = 56), neurological disorder (8% *n* = 45), cancer (7%, *n* = 37), and immune deficiency (2%, *n* = 13). There were no statistically significant differences in any of the clinical characteristics of the patients that consented for the BALI study who did, or did not, have plasma IL-8 or IL-8 genetic variants tested. Mortality in the BALI cohort was 9%, with a median duration of mechanical ventilation of 7.1 days (IQR, 4.0–13.6) and a median PICU length of stay in survivors of 10.6 days (IQR, 6.6–18.4).

Plasma IL-8 levels were highest on the day of intubation (day 0) and tapered over the days to follow with a statistically significant difference in median values across the indicated days (*p* < 0.001, Table 1). There was no association between plasma IL-8 level and age or gender (data not shown). IL-8 was significantly elevated in those with a past medical history of cancer (*p* < 0.05 on each day) or immune deficiency (*p* < 0.05 on days 1, 2, 3) as described in Additional file 1. IL-8 was also significantly higher in children with a primary diagnosis of sepsis (Additional file 1: Figure S1). The frequency of a primary diagnosis of sepsis was 38% and 41% in those with a history of immune deficiency or cancer, respectively.

**Table 1:** Plasma interleukin-8 levels decrease over time

Day	*N*	Mean (SD)	Median (range)
0	57	759 (1797)	54 (4, 7373)
1	239	344 (1035)	44 (0, 6900)
2	362	252 (872)	39 (0, 8162)
3	325	221 (822)	37 (0, 7706)

IL-8 levels are shown as picogram per milliliter. Day 0 is the day of intubation. The *n* is the number of plasma samples analyzed. Plasma IL-8 levels were log transformed for analysis due to skewness. Medians are significantly different across days, *p* < 0.001 using generalizing estimating equations to account for correlations within individuals

### Plasma IL-8 and PARDS development

**Fig. 1:**
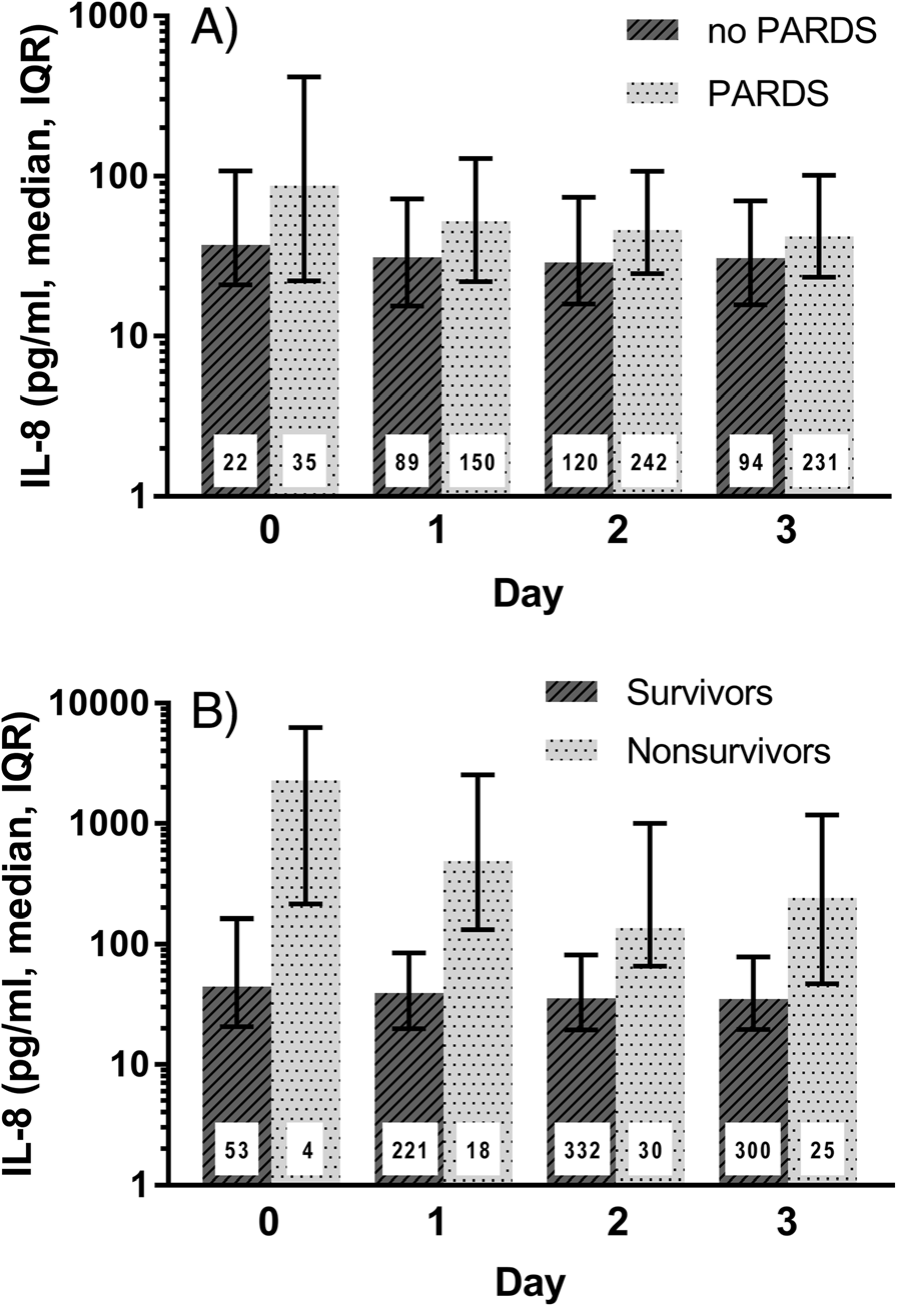
Plasma interleukin-8 is higher in children with pediatric acute respiratory distress syndrome and in non-survivors. **a** Comparison of plasma IL-8 in children with or without PARDS. The group with PARDS included children meeting the criteria for PARDS on the indicated day or any previous day. Plasma IL-8 was significantly higher in patients with PARDS across all days (*p* < 0.0001 using GEE methods as described in the “Methods” section), but when examining each day only achieved significance on day 2 (*p* = 0.02). **b** Comparison of plasma IL-8 in children who died or survived. IL-8 is significantly higher in non-survivors on each day (*p* < 0.05). Day 0 is the day of intubation. The number inside each bar indicates the number of plasma samples analyzed from the indicated days. IL-8 interleukin-8, PARDS pediatric acute respiratory distress syndrome

Of the 549 patients enrolled in BALI, 69% (*n* = 378) developed PARDS. Within the PARDS cohort, 83% met the diagnosis for PARDS at the time of intubation (day 0) and 94% by day 1. Plasma IL-8 levels were higher in children with PARDS compared to those children without PARDS on all days measured, but when examining each day, achieved statistical significance only on day 2 (*p* = 0.02 on day 2, Fig. 1a). Multivariable analyses adjusted for age, PRISM III, and sepsis diagnosis demonstrated that plasma IL-8 was not independently associated with PARDS across all days or on any specific day (Additional file 1: Table S3).

### Analyses of plasma IL-8 and relevant clinical outcomes

**Fig. 2:**
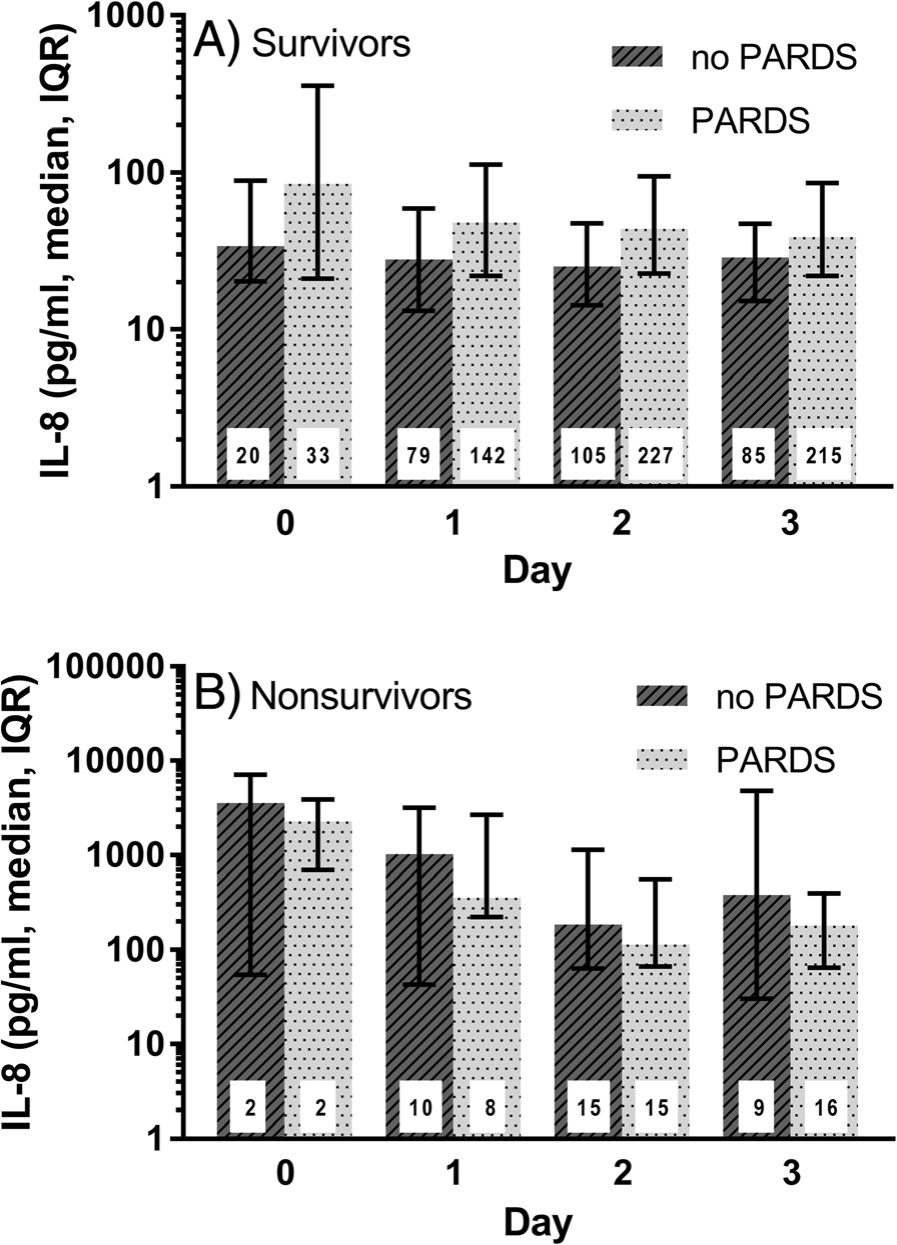
Comparison of plasma interleukin-8 levels in survivors and non-survivors with or without pediatric acute respiratory distress syndrome. **a** Comparison of plasma IL-8 in survivors with acute respiratory failure with or without PARDS. IL-8 is significantly higher in survivors with PARDS on each day (*p* < 0.05). **b** Comparison of plasma IL-8 in non-survivors with acute respiratory failure with or without PARDS. In non-survivors, IL-8 levels are not significantly different in children with or without PARDS. Day 0 is the day of intubation. The number inside each bar indicates the number of plasma samples analyzed from the indicated days. IL-8 interleukin-8, PARDS pediatric acute respiratory distress syndrome

On bivariate analysis of this cohort of children with acute respiratory failure, plasma IL-8 was significantly higher in non-survivors on each day measured (Fig. 1b, *p* < 0.05) with median IL-8 levels 4–12 fold higher in non-survivors than survivors. As IL-8 has been reported to be associated with death in children with PARDS, the cohort was stratified by PARDS to examine whether PARDS patients were responsible for the higher IL-8 levels observed in non-survivors. As shown in Fig. 2a, the levels of IL-8 were significantly higher in survivors with PARDS compared to survivors without PARDS; however, the levels of IL-8 were not significantly different in non-survivors with and without PARDS **(**Fig. 2b) but are higher than those seen in survivors with or without PARDS (Fig. 2b vs a) suggesting that IL-8 is associated with death in patients with acute respiratory failure independent of whether the child has PARDS. On multivariate analysis with adjustment for severity of illness (PRISM III), age, and sepsis diagnosis, plasma IL-8 was independently associated with death on days 1, 2, and 3 (Table 2). When PARDS was added to the model, IL-8 was still independently associated with death (Additional file 1: Table S4) again suggesting that IL-8 is associated with death both in the whole cohort and in patients without PARDS.

**Table 2:** Multivariable analysis of association of interleukin-8 with death

Day	Variable	Odds ratio	95% Confidence interval	*p* ^a^
0	IL-8	2.05	0.56–7.46	0.28
	Age	1.00	0.83–1.20	0.99
	PRISM III	0.96	0.68–1.31	0.73
	Sepsis	1.79	0.27–12.1	0.55
1	IL-8	2.33	1.51–3.60	< 0.001
	Age	1.14	1.04–1.25	0.007
	PRISM III	0.97	0.88–1.06	0.50
	Sepsis	0.76	0.20–2.99	0.70
2	IL-8	1.90	1.39–2.60	< 0.001
	Age	1.13	1.06–1.22	< 0.001
	PRISM III	1.00	0.93–1.08	0.96
	Sepsis	1.09	0.38–3.13	0.88
3	IL-8	2.19	1.48–3.24	< 0.001
	Age	1.14	1.06–1.22	< 0.001
	PRISM III	0.98	0.91–1.06	0.58
	Sepsis	0.87	0.24–3.11	0.83

^a^*p* value using a multivariable logistic regression model with adjustment for covariates. The *n* for days 0–3 is 57, 239, 362, and 325, respectively. Day 0 is the day of intubation. IL-8 interleukin-8, PRISM pediatric risk of mortality

**Fig. 3:**
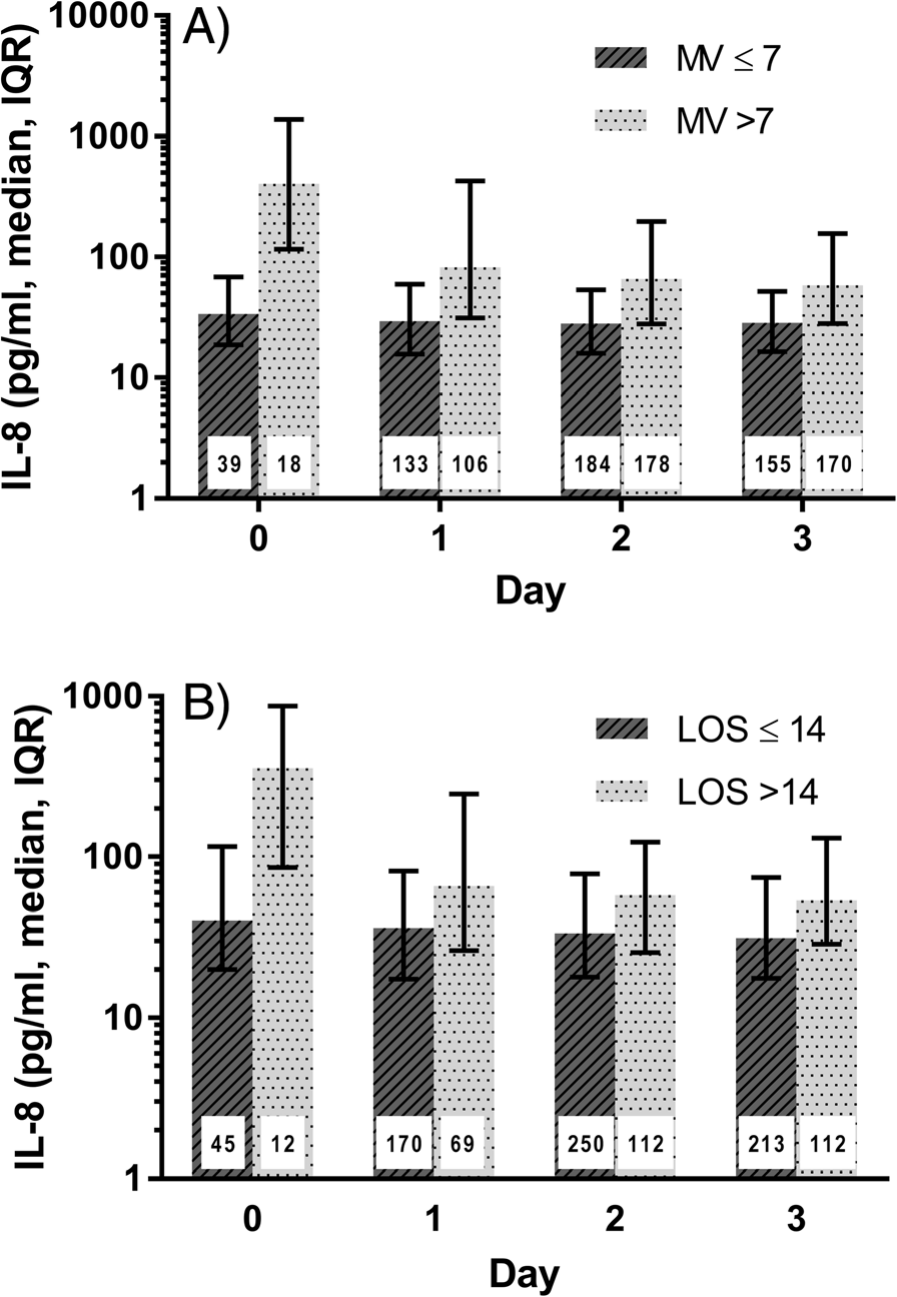
Plasma interleukin-8 is higher in children with a longer duration of mechanical ventilation or length of stay. **a** Comparison of plasma IL-8 in children with acute respiratory failure who were mechanically ventilated for ≤ 7 days to those mechanically ventilated for > 7 days; *p* < 0.01 on each day. **b** Comparison of plasma IL-8 in children with acute respiratory failure who were in the PICU for ≤ 14 days to those in the PICU for > 14 days; *p* < 0.01 on each day. Day 0 is the day of intubation. The number inside each bar indicates the number of plasma samples analyzed from the indicated days. IL-8 interleukin-8, LOS length of stay in the PICU, MV mechanical ventilation, PICU pediatric intensive care unit

Bivariate analysis also indicated that plasma IL-8 was significantly correlated with duration of mechanical ventilation on each day measured and with PICU length of stay on days 1, 2, and 3 (Additional file 1: Table S5). When duration of mechanical ventilation was dichotomized into 7 or more ventilator days versus fewer than 7 days (median duration of mechanical ventilation was 7.1 days, IQR, 4.0–13.6), plasma IL-8 was significantly higher in patients with a duration of mechanical ventilation ≥ 7 days on each day measured (*p* < 0.01, Fig. 3a). Using 14 days to dichotomize PICU length of stay (median PICU length of stay was 10.6 days, IQR, 6.6–18.4), IL-8 was significantly elevated on each day measured in those patients requiring a prolonged PICU stay (14 or more days vs fewer than 14 days, *p* < 0.01 on each day, Fig. 3b). Using Poisson regression and controlling for age, severity of illness, and sepsis diagnosis, plasma IL-8 was independently associated with prolonged duration of mechanical ventilation on each day measured (*p* ≤ 0.01 on each day). Finally, plasma IL-8 was associated with prolonged PICU length of stay in survivors on each day measured when adjusted for age and severity of illness and was also associated with longer length of stay on days 0 (*p* = 0.01), 2 (*p* = 0.02), and 3 (*p* = 0.03) when sepsis diagnosis was also added to the model (data not shown).

### Genetic association studies for IL-8

Three IL-8 SNPs previously reported to be associated with severity in cystic fibrosis (rs4073, rs2227306, rs2227307) or with the length of mechanical ventilation in adults with ARDS (rs4073) were examined for association with PARDS. The cohort was stratified into non-Hispanic Caucasians, Hispanic Caucasians, and African Americans for analyses which were done with adjustment for population structure (as described under the “Methods” section). None of the variants examined showed a significant association with PARDS within any of the subgroups or when the three subgroups were combined in a meta-analysis (*n* = 412). In addition, these variants were not associated with plasma levels of IL-8.

## Discussion

Identification of children within the at-risk population with a higher likelihood of developing PARDS using either specific biomarker levels and/or specific genetic variations provides a unique insight into PARDS pathogenesis and may identify novel therapeutic targets allowing future studies to stratify patients so that they might benefit from targeted therapies. This is the first study in children examining whether IL-8 is associated with the development of PARDS. Plasma IL-8 is significantly elevated in pediatric acute respiratory failure and was even higher in those who develop PARDS; however, multivariable analysis adjusting for relevant covariates indicates that plasma IL-8 is not independently associated with PARDS.

Our data indicate a very strong association of elevated IL-8 levels with mortality in this cohort of children with acute respiratory failure independent of whether the child had PARDS. IL-8 levels were not significantly different in non-survivors with or without PARDS. Plasma IL-8 levels in non-survivors are 4–12 fold higher than in survivors, and this elevation is present for several days after onset of acute respiratory failure. Further, the association of IL-8 remained independently associated with death even after adjusting for many key covariates: severity of underlying disease (PRISM III), sepsis diagnosis, development of PARDS, and others. Plasma IL-8 elevation was also strongly associated with prolonged mechanical ventilation, PICU length of stay, and degree of pulmonary morbidity (as measured by oxygenation defect).

Persistent elevation of IL-8 has been reported in adults with ARDS and also appears to be related to the differential impact of ventilator strategy or steroid responsiveness in randomized controlled trials of adult ARDS patients [23–25]. To date, pediatric studies have largely focused on IL-8 elevations at the onset of disease [8, 26]. The persistence of inflammation after the onset of pediatric acute respiratory failure in general, and in PARDS specifically, offers a unique opportunity to determine whether measuring plasma IL-8 over time can be used as an indicator of therapeutic effectiveness and/or disease progression. If so, real-time IL-8 measurement to guide management strategies in randomized clinical trials, as well as routine clinical practice, appears promising if technological advances allowing the measurement of cytokines in real-time were available.

IL-8 levels were highly variable, especially on the day of intubation (day 0) where levels ranged from 4 to 7373 pg/ml. This degree of variability may have profound implications for future therapeutic trials targeting the inflammatory cascade in either pediatric respiratory failure and/or PARDS, particularly since elevated IL-8 is one of the biomarkers shown to be able to distinguish between the hyperinflammatory and hypoinflammatory subphenotypes in adults ARDS [17, 27, 28]. The observed degree of variability in IL-8 levels together with adult data strongly suggest the need for future studies to determine whether subphenotypes are also present in PARDS patients and whether subphenotypes differ between children and adults.

Although age does impact the development of the immune system and levels of cytokines have been reported to vary with age, in this cohort of children with acute respiratory failure and/or PARDS, plasma IL-8 was independent of age. Indeed, age was not associated with plasma IL-8 levels on any day measured. This lack of an association of age with IL-8 levels may be because these children are critically ill and in the midst of a very marked inflammatory response and because of the high degree of variability. The lack of an impact of age on the association of IL-8 levels with these outcome measures underscores important similarities between the inflammatory pathophysiology exhibited in ARDS in adult and pediatric patients. Our data support the inclusion of both adult and pediatric patients into future trial design aimed at impacting the inflammatory cascades during early ARDS. However, it remains imperative for any biological marker which will potentially be used for bedside risk stratification for clinical or research purposes to be tested for associations with age and to be evaluated in both adults and children.

Our study has some limitations. The definition of PARDS was changed during the study enrollment period. For the analysis, PARDS was defined using the more recent PALICC criteria. However, the parent RESTORE trial and the BALI study were designed using AECC criteria to diagnose ARDS; consequently, chest radiograph interpretation was limited to the presence or absence of new bilateral infiltrates on each study day. Lastly, while the majority of patients met the criteria for PARDS on the day of intubation (day 0), only 16% of patients had blood drawn for plasma biomarkers on day 0. This is noteworthy as it limited our ability to assess whether plasma IL-8 was higher before the onset of PARDS in patients who went on to develop PARDS. As a result, we were unable to determine whether IL-8 levels are significantly higher in those who do not meet the criteria for PARDS but are destined to develop PARDS subsequently.

## Conclusions

In conclusion, plasma IL-8 is remarkably elevated over several days after the onset of acute respiratory failure and PARDS. These elevations are robust and are independently associated with multiple relevant clinical outcomes, most importantly death, but are not independently associated with PARDS. The wide range of plasma IL-8 seen in our investigation suggests the potential existence of subphenotypes in children with PARDS which should be investigated further as subphenotypes can significantly impact future therapeutic trial design. Additional investigation is warranted to determine the utility of serial IL-8 measurement in corroborating disease progression and/or treatment effectiveness in pediatric acute respiratory failure and PARDS.

## Additional file

Additional file 1: Supplementary tables and figures for a prospective investigation of interleukin-8 levels in pediatric acute respiratory failure and acute respiratory distress syndrome. Supplementary tables (S1–S5) and figures (S1–S2) are found in the additional file. (DOCX 273 kb)

## Abbreviations

AECC: American European Consensus Conference
ARDS: Acute respiratory distress syndrome
FiO_2_: Fraction of inspired oxygen
GEE: Generalized estimating equations
IL-8: Interleukin-8
OI: Oxygenation index
OSI: Oxygen saturation index
P_a_O_2_: Partial pressure of oxygen
PARDS: Pediatric acute respiratory distress syndrome
PICU: Pediatric intensive care unit
PRISM: Pediatric risk of mortality
SNP: Single nucleotide polymorphism
TNF: Tumor necrosis factor
