# Supplementary material for: A prospective investigation of interleukin-8 levels in pediatric acute respiratory failure and acute respiratory distress syndrome
Source: Crit Care. 2019 Apr 17;23:128. doi: 10.1186/s13054-019-2342-8 (PMC6471952; doi:10.1186/s13054-019-2342-8)
Supplement: Supplementary file 1 — Supplementary tables and figures for a prospective investigation of interleukin-8 levels in pediatric acute respiratory failure and acute respiratory distress syndrome. Supplementary tables (S1–S5) and figures (S1–S2) are found in the additional file. (DOCX 273 kb) [file 13054_2019_2342_MOESM1_ESM.docx]

**Additional File 1**

**A Prospective Investigation of Interleukin-8 Levels in Pediatric Acute Respiratory Failure and Acute Respiratory Distress Syndrome**

Heidi Flori, MD^1^, Anil Sapru, MD^2^, Michael W. Quasney, MD^1^, Ginny Gildengorin, PhD^3^, Martha A.Q. Curley, PhD^4,5^, Michael A. Matthay, MD^6^, and Mary K. Dahmer, PhD^1^* for the BALI and RESTORE Study Investigators and Pediatric Acute Lung Injury and Sepsis Investigators (PALISI) Network

1. Division of Pediatric Critical Care Medicine, Department of Pediatrics and Communicable Diseases, University of Michigan, 1500 East Medical Center Dr, F6790/5243, Ann Arbor, MI 48109, USA

2. Department of Pediatrics, University of California, Los Angeles, CA, USA

3. Children’s Hospital Oakland Research Institute, UCSF Benioff Children’s Hospitals, Oakland, CA, USA

4. Department of Family and Community Health (School of Nursing), Division of Anesthesia and Critical Care Medicine (Perelman School of Medicine) University of Pennsylvania, Philadelphia, PA, USA

5. Research Institute; Children’s Hospital of Philadelphia, Philadelphia, PA, USA

6. Departments of Medicine and Anesthesia, Cardiovascular Research Institute, University of California, San Francisco, CA, USA

Corresponding Author: Mary K. Dahmer, PhD

mkdahmer@umich.edu

734-647-1426

Fax: 734-232-4683

***Plasma IL-8 Levels and Association with Primary Diagnoses and Clinical Covariates***

As expected, there were differences in IL-8 levels across primary diagnoses (Figure S1, p < 0.001 for days 1, 2, or 3). The difference in IL-8 across different primary diagnoses appear to be driven by higher levels of plasma IL-8 in patients with sepsis compared to those with a primary diagnosis of pneumonia, asthma, bronchiolitis or other (p<0.05 for each comparison on days 1, 2, or 3) and lower levels of IL-8 in patients with a primary diagnosis of asthma compared to those with a primary diagnosis of pneumonia or other (p<0.05 for each comparison on days 1, 2, or 3). When categorized as infectious, non-infectious and indeterminate, again statistically significant differences were achieved on days 1, 2 or 3 (p < 0.05, Figure S2).

There was no association between plasma IL-8 level and age, gender, or treatment group assigned to in the clinical trial (data not shown). Evaluation of plasma IL-8 and co-morbid conditions yielded no association of the biomarker with past medical history of prematurity, aspiration pneumonia or seizure disorder. IL-8 was significantly elevated on each day in those with past medical history of cancer (p < 0.05 for each day) or immune-compromise (days 1, 2 and 3, p < 0.05). However, the frequency of a primary diagnosis of sepsis, which also was associated with higher IL-8 levels (see above), was higher in those with a past medical history of cancer (n=37) or immune-compromise (n=13), than in children without those conditions (38% vs 18%, p=0.08, and 41% vs 17%, p=0.0005, respectively). In addition, 5 of the 13 with a history of immune-compromise also had a history of cancer. Interestingly, IL-8 was significantly lower on days 0,1, 2 or 3 in patients with history of asthma (p< 0.05) compared to other co-morbidities, ~50% of these children had a primary diagnosis of asthma which also had significantly lower IL-8 levels.

Plasma IL-8 correlated with oxygenation defect as measured by both PaO_2_/FiO_2_ (day 0 and 1, p < 0.01) and oxygenation index (OI) (on each day, p < 0.05, see Table S1) and severity of illness as measured by PRISM III score (on each day, p < 0.001, see Table S2).

| Table S1. Plasma Interleukin-8 Levels are Correlated with Indices of Oxygenation in Patients with Acute Respiratory Failure | | | | | |
| --- | --- | --- | --- | --- | --- |
| **Day** | **N** | **Oxygenation Index**  **Correlation Coefficient** | **p** | **PaO_2_/FiO_2_**  **Correlation Coefficient** | **p** |
| 0 | 41 | 0.37 | 0.02 | -0.41 | 0.008 |
| 1 | 171 | 0.39 | <0.001 | -0.31 | <0.001 |
| 2 | 253 | 0.30 | < 0.001 | -0.11 | 0.08 |
| 3 | 220 | 0.24 | <0.001 | -0.10 | 0.15 |
| N indicates the number of plasma samples analyzed from the indicated day with oxygenation index or PaO_2_/FiO_2_ available on that day. | | | | | |

| Table S2. Plasma Interleukin-8 Levels are Correlated with PRISM III Score | | | |
| --- | --- | --- | --- |
| **Day** | **N** | **Correlation Coefficient** | **p** |
| 0 | 57 | 0.55 | <0.001 |
| 1 | 238 | 0.43 | <0.001 |
| 2 | 360 | 0.43 | < 0.001 |
| 3 | 323 | 0.36 | <0.001 |
| N is number of plasma samples analyzed from the indicated day. PRISM=pediatric risk of mortality | | | |

| **Table S3. Multivariable Analysis of Association of Interleukin-8 with Pediatric Acute Respiratory Distress Syndrome** | | | | |
| --- | --- | --- | --- | --- |
| **Day** | **Variable** | **Odds Ratio** | **95% Confidence Interval** | **p** |
| 0 | IL-8 | 1.13 | 0.75-1.72 | 0.56 |
|  | Age | 0.97 | 0.88-1.08 | 0.59 |
|  | PRISM III | 1.02 | 0.92-1.13 | 0.77 |
|  | Sepsis | 2.54 | 0.55-11.8 | 0.23 |
| 1 | IL-8 | 1.12 | 0.90-1.38 | 0.32 |
|  | Age | 1.01 | 0.97-1.06 | 0.57 |
|  | PRISM III | 1.02 | 0.98-1.07 | 0.33 |
|  | Sepsis | 0.93 | 0.46-1.88 | 0.83 |
| 2 | IL-8 | 1.21 | 0.98-1.49 | 0.08 |
|  | Age | 1.01 | 0.97-1.05 | 0.78 |
|  | PRISM III | 1.01 | 0.98-1.05 | 0.48 |
|  | Sepsis | 0.88 | 0.48-1.60 | 0.67 |
| 3 | IL-8 | 1.18 | 0.92-1.51 | 0.20 |
|  | Age | 1.00 | 0.96-1.04 | 0.88 |
|  | PRISM III | 1.02 | 0.98-1.06 | 0.26 |
|  | Sepsis | 0.72 | 0.37-1.41 | 0.34 |
| N for Days 0-3 is 57, 238, 360, and 323, respectively. PRISM=pediatric risk of mortality | | | | |

| **Table S4. Multivariable Model of Association of Interleukin-8 with Death Adjusting for Pediatric Acute Respiratory Distress Syndrome** | | | | |
| --- | --- | --- | --- | --- |
| **Day** | **Variable** | **Odds Ratio** | **95% Confidence Interval** | **p** |
| 0 | IL-8 | 1.05 | 0.98-1.13 | 0.15 |
|  | Age | 1.00 | 0.99-1.01 | 0.92 |
|  | PRISM III | 1.00 | 0.98-1.02 | 0.86 |
|  | PARDS | 0.94 | 0.81-1.08 | 0.35 |
| 1 | IL-8 | 1.06 | 1.02-1.09 | 0.002 |
|  | Age | 1.01 | 1.00-1.01 | 0.02 |
|  | PRISM III | 1.00 | 0.99-1.01 | 0.90 |
|  | PARDS | 0.92 | 0.86-0.99 | 0.03 |
| 2 | IL-8 | 1.05 | 1.02-1.08 | <0.001 |
|  | Age | 1.01 | 1.00-1.01 | <0.001 |
|  | PRISM III | 1.00 | 1.00-1.01 | 0.47 |
|  | PARDS | 0.92 | 0.86-0.98 | 0.006 |
| 3 | IL-8 | 1.05 | 1.02-1.09 | 0.002 |
|  | Age | 1.01 | 1.00-1.01 | 0.001 |
|  | PRISM III | 1.00 | 1.00-1.00 | 0.98 |
|  | PARDS | 0.96 | 0.90-1.02 | 0.17 |
| N for Days 0-3 is 57, 239, 362, and 325, respectively. PRISM=pediatric risk of mortality, PARDS=pediatric acute respiratory distress syndrome | | | | |

| Table S5. Plasma Interleukin-8 Levels are Correlated with Duration of Mechanical Ventilation and PICU Length of Stay | | | | | | |
| --- | --- | --- | --- | --- | --- | --- |
| **Day** | **N^a^** | **Duration of MV**  **Correlation Coefficient** | **p** | **N^b^** | **PICU LOS**  **Correlation Coefficient** | **p** |
| 0 | 57 | 0.33 | 0.01 | 53 | 0.19 | 0.17 |
| 1 | 239 | 0.26 | <0.001 | 221 | 0.16 | 0.02 |
| 2 | 362 | 0.19 | < 0.001 | 332 | 0.18 | < 0.001 |
| 3 | 325 | 0.20 | <0.001 | 300 | 0.20 | < 0.001 |
| PICU LOS analysis was performed in survivors. N indicates either: ^a^the number of plasma samples analyzed from the indicated day, or ^b^the number of plasma samples from survivors analyzed from the indicated day. MV=mechanical ventilation, LOS=length of stay, PICU=pediatric intensive care unit | | | | | | |

**Figure S1. Plasma IL-8 levels by primary diagnoses.**

Plasma IL-8 levels in patients with the most common primary diagnoses. IL-8 levels are significantly different across primary diagnoses on Days 1, 2, or 3, (p<0.001* for each day). Plasma IL-8 is significantly higher in patients with sepsis compared to those with a primary diagnosis of pneumonia, asthma, bronchiolitis or aspiration (**p<0.05 for each comparison on Days 1, 2, or 3) and significantly lower in patients with a primary diagnosis of asthma compared to those with a primary diagnosis of pneumonia, sepsis or other (**p<0.05 for each comparison on Days 1, 2, or 3). On Day 3 plasma IL-8 is also significantly higher in those with sepsis than in those with a primary diagnosis of other. On Day 1 and 3 plasma IL-8 in patients with a primary diagnosis of asthma is significantly lower than in those with a primary diagnosis of aspiration. *significance determined using an analysis of variance; **significance determined using a Tukey-Kramer post-test adjusting for multiple comparisons. The number within the bars is the number of patients with that diagnosis with IL-8 measured on that study day. IL-8=interleukin-8

**Figure S2. Plasma IL-8 levels when primary diagnosis is** **grouped as infectious, non-infectious or indeterminant.**

Plasma IL-8 levels in patients grouped as infectious, non-infectious or indeterminant grouped as defined under Methods. Significance was determined by an analysis of variance. IL-8 = interleukin-8
